# Retraction: Deficient for endoplasmic reticulum calcium sensors Stim1 and Stim2 affects aberrant antibody affinity maturation in B cells

**DOI:** 10.18632/oncotarget.27171

**Published:** 2019-08-20

**Authors:** Xuhua Mao, Jianfeng Zhang, Yue Han, Chao Luan, Yu Hu, Zhimin Hao, Min Chen

**Affiliations:** ^1^ Department of Clinical Laboratory, Yixing People’s Hospital, China; ^2^ Department of Preventive Health Care, the Second Affiliated Hospital of Southeast University, China; ^3^ Jiangsu Key Laboratory of Molecular Biology for Skin Diseases and STIs, Institute of Dermatology, Chinese Academy of Medical Sciences, China

**This article has been retracted:** After re-analyzing the original data for this article, the authors found discrepancies between the published data and the ones recently analyzed. This raises serious concerns about the accuracy of the data. The authors believe that further research is needed to confirm these findings, and are no longer confident about some of the data published in the original publication. As a result, all authors have agreed to the retraction of this paper from Oncotarget.

Original article: Oncotarget. 2016; 7:60885–60895. 60885-60895 . https://doi.org/10.18632/oncotarget.11659

